# Global Trends in Research of Lipid Metabolism in T lymphocytes From 1985 to 2022: A Bibliometric Analysis

**DOI:** 10.3389/fimmu.2022.884030

**Published:** 2022-05-26

**Authors:** Peng Chen, Cheng Zhong, Shengxi Jin, Yiyin Zhang, Yirun Li, Qiming Xia, Jiaxi Cheng, Xiaoxiao Fan, Hui Lin

**Affiliations:** ^1^ Department of General Surgery, Sir Run Run Shaw Hospital, School of Medicine, Zhejiang University, Hangzhou, China; ^2^ College of Biomedical Engineering and Instrument Science, Zhejiang University, Hangzhou, China

**Keywords:** lipid metabolism, T lymphocytes, Bibliometric, Citespace, VOSviewer

## Abstract

Lipids are involved in both energy metabolism and signaling transduction. Abnormal lipid metabolism in T cells is associated with the differentiation, longevity and activity of T cells, which has received increasing concern since its firstly reported in 1985. To evaluate the trends of lipid metabolism in T cells and map knowledge structure, we employed bibliometric analysis. A total of 286 related publications obtained from the Web of Science Core Collection published between 1985 and 2022 were analyzed using indicators of publication and citation metrics, countries, institutes, authors, cited references and key words. The present research status, the global trends and the future development directions in lipid metabolism and T cells were visualized and discussed. In summary, this study provides a comprehensive display on the field of lipid metabolism in T cells, which will help researchers explore lipid metabolism in T cells more effectively and intuitively.

## Introduction

T lymphocytes (also named T cells) are the main components of lymphocytes, which derived from bone marrow pluripotent stem cells. Immunotherapy based on T cells has been confirmed as a successful method for the treatment of various diseases (1–3). The differentiation, longevity, durability, and functionality of T cells play vital roles in regulating the efficacy of immunotherapy, which are largely determined by the metabolic activity of glucose, lipid and amino acid (4, 5). Clarifying the metabolic status in T cells can help to control T cell differentiation and fate, so that the effect of immunotherapy based on T cells can be amplified.

Lipids, consisting of acylglycerols, isoprenoids, sterols, and phospholipids, etc., are hydrophobic biomolecules making up biological membranes (6, 7). Besides participating in energy metabolism, lipids on the membranes can also give rise to signaling transduction in response to extracellular stimuli through working as second messengers, highlighting the importance of lipid components on membrane (8, 9). As signaling molecules, lipid metabolism in cells, including biosynthesis, storage and degradation, participates in controlling the status and function of various cells, including T cells. Actually, the correlation between lipid metabolism and T cells has received limited but increasing concern since related publication firstly reported in 1985 (9). Till now, the effect and status of lipid metabolism has been partly revealed in regulating the differentiation, longevity and activity of T cells. For instance, Pearce et al. (10) has demonstrated that the deficiency of mitochondrial long chain fatty acid oxidation (LC-FAO) caused by specific deletion of TNF receptor associated factor 6 (TRAF6) in T cells results in defect in their ability to generate long lived memory T (T_M_) cells, highlighting that LC-FAO is indispensable to the formation of T_M_ cells. Opposite results indicated that LC-FAO is not required for T_M_ cell formation in both human and mice (11). These contradictory results suggested the complicated and vital effect of lipid metabolism in T cells. However, limited studies have been published in this field, which means that researches on lipid metabolism in T cells is still in development stage. Analyzing what has been achieved in this field can help us to estimate the developmental trend in lipid metabolism in T cells and guide experimentation strategies and funding decisions.

Bibliometrics is a method exploring library and information science through comprehensively analyzing the bibliographic material based on quantitative measurement (12). Bibliometrics can help researchers quickly grasp the hotspots and development trends in their fields, laying a cornerstone for the direction of future researched (13, 14). Over the years, the outcomes of application of bibliometrics in immune cells are fruitful (15–17). However, bibliometric study on the relationship between T cells and lipid metabolism is still a void. Therefore, we here performed a bibliometrics study on lipid metabolism in T cells based on data published to help to make further decisions in this field.

## Materials and Methods

### Data Sources and Search Strategies

**Figure 1:**
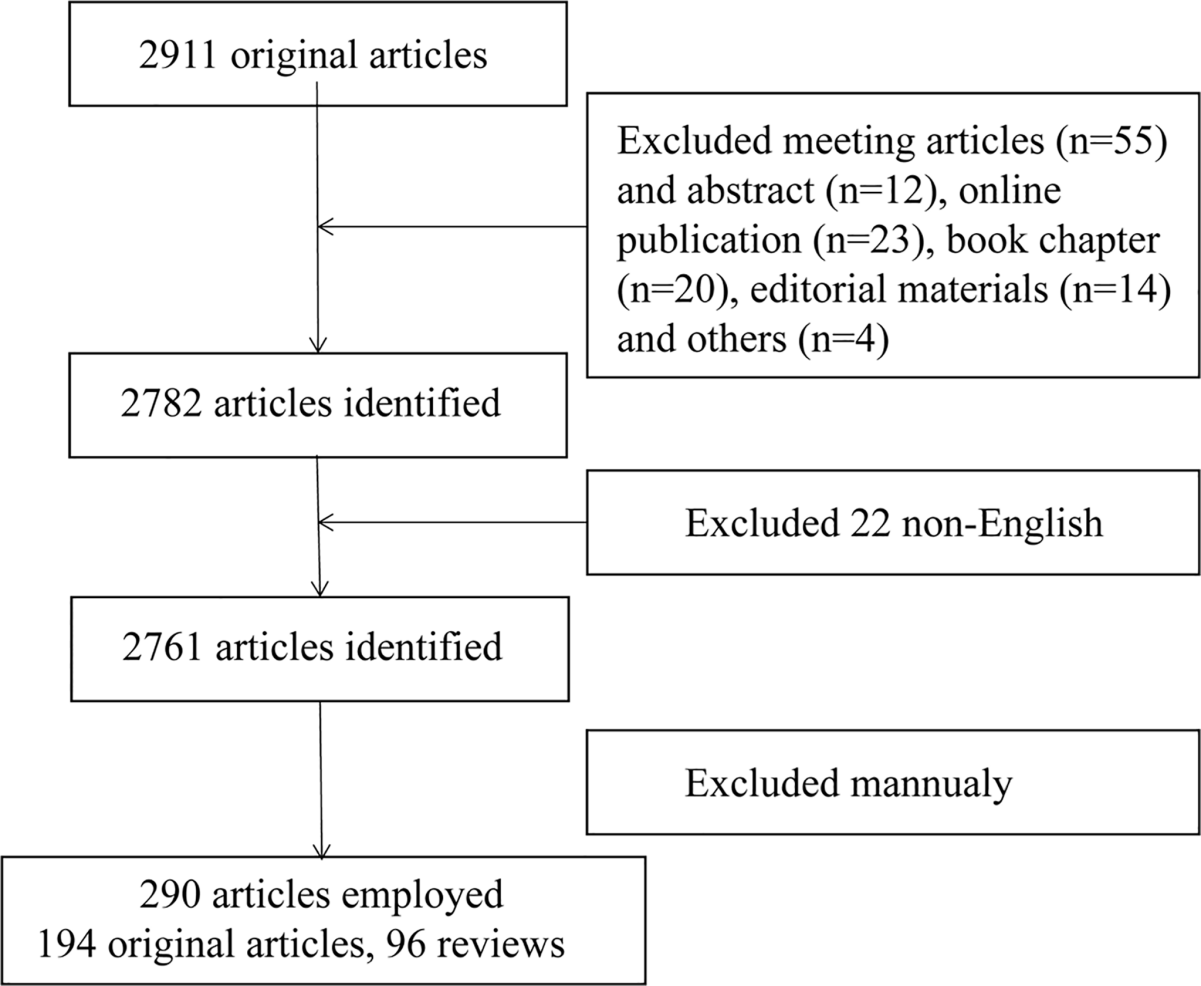
Flowchart of the screening process.

The Web of Science Core Collection (WoSCC) was used to search and obtain data on T cells and lipid metabolism in the past 40 years (from 1985 to 2022). Literature retrieval was conducted within one day (February 19, 2022) to avoid fluctuations in citations caused by rapid updates of publications. The search formula was set to TS= (T lymphocytes OR T cells) AND TS=(lipid metabolism). A total of 2911 studies were acquired *via* this step. Next, 128 articles including meeting articles (n=55), online publication (n=23), book chapter (n=20), editorial materials (n=14), meeting abstract (n=12) and others (n=4) were excluded. Further, only articles written in English was kept (n=2761). Finally, we read the title, abstract or even full text of these publications to screen out papers closely related to the topic we studied (lipid metabolism in T cells). The topics we mainly focus on are how T cells recognized extracellular lipid, how lipid metabolism regulated the fate of T cells and how lipid metabolism in T cells was regulated. Finally, only 290 publications were enrolled for bibliometrics analysis. The detailed process was shown in **Figure 1**. The studies used included the following information: the number of publications and citations, titles, publication year, countries/regions, affiliations, authors, journals, key words and references. This procedure was conducted by three researchers (PC, CZ and SJ) independently and any potential differences were discussed. The publications list of the 290 articles was provided in **Supplementary Table 1**.

### Statistical Analysis

All valid data were imported to Microsoft Office Excel 2019, HisCite (version 2009.08.24), VOSviewer (version 1.6.18), CiteSpace (version 5.8.R2), and Bibliometrix 4.1.0 Packages based on the R language to perform visual analysis.

Microsoft Office Excel 2019 was employed to plot radar charts.

HisCite (18) was employed to calculate the number of publications, total local citation score (TLCS) and total global citation score (TGCS) for each publication year, and top countries, authors, journals and institutions.

Vosviewer (19) was hired to visualize the bibliometric network including the cooperation among countries and institutions. The colors of the nodes represent various times or clusters; the size of the nodes means the number of publication; the thickness of the line represents the strength of the relation.

CiteSpace (20) was used to conduct cluster analysis and bursts of references and keywords, timeline views. Cluster analysis of key words can identify vital areas on lipid metabolism in T cells through classifying keywords and references. The modularity Q > 0.3 and mean silhouette > 0.5 indicated the clustering results are enough and convincing. Keywords and references bursts can be used to detect new research trends on lipid metabolism in T cells.

The Bibliometrix Packages (21) is a tool based on the R language used for bibliometric analysis, which was used to analyze the annual growth rate of publications and top 10 cited references here.

## Results

### Analysis of the Overall Distribution

**Figure 2:**
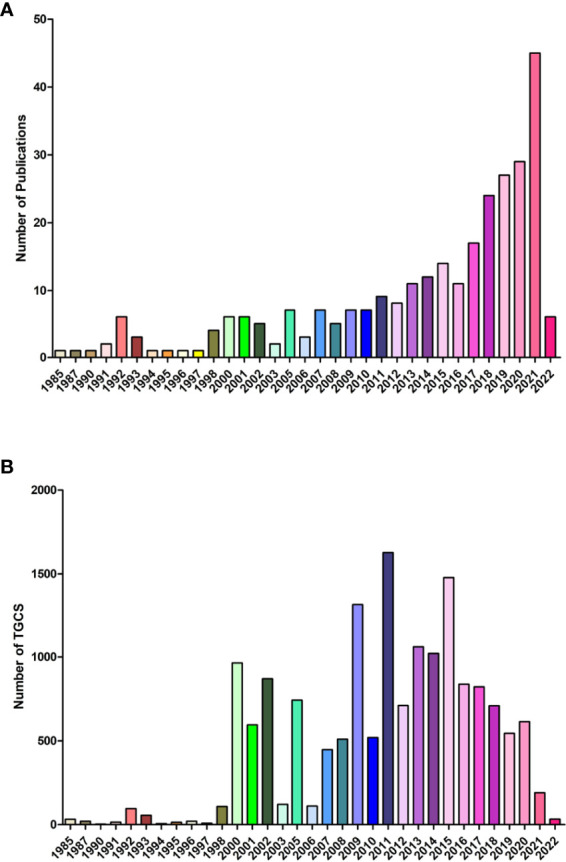
Overall distribution of publication outputs on lipid metabolism in T cells **(A)** Global annual output trends; **(B)** Global annual citation.

A total of 194 original researches and 96 reviews associated with lipid metabolism in T cells were screened out. Curve fitting analysis (**Figure 2A**) revealed an overall increasing trend of the annual amount of publications on lipid metabolism in T cells since its first reported in 1985, and the annual growth rate is 3.39%. During 1985 to 2012, less than 10 publications on lipid metabolism in T cells was published annually. Specially, in the earliest six years (1985-1990), only three papers were published in 1985 (written by Goppelt M), 1987 (written by Cockcroft S) and 1990 (written by Stephen FD), respectively. Goppelt et al. (9) proposed the effect of lipid metabolism in the activation of T cells for the first time. Closed to the same time, Cockcroft et al. (22) identified activated inositol lipid kinase as a main signal in the activation of human T cells. After that, Stephen et al. (23) identified the effect of free fatty acids on spectrin organization in lymphocytes. Afterwards, the number of publications gradually increased from 11 in 2013 to 45 in 2021. Although the number of the papers is still not very high, the average annual growth increased rapidly. Especially, the amount of papers in 2021 (n=45) was even slightly higher than that in the earliest 20 years (1985-2003, n=41). Although the annual citation of publications fluctuated over the past years, an overall increasing trends could still been found (**Figure 2B**). The increased annual publication and citation highlighted the rapidly progression of interest in the field of lipid metabolism in T cells.

### Analysis of Countries/Regions

**Figure 3:**
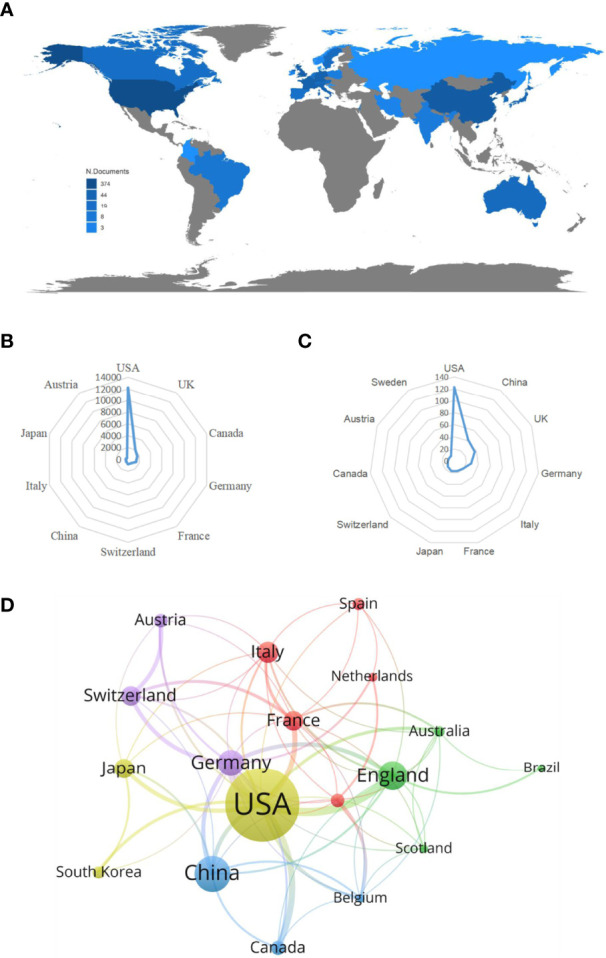
Analysis of countries/regions. **(A)** Geographical distribution of global output; **(B)** Radar map of the top 10 productive countries; **(C)** Radar map of TGCS of the top 10 productive countries; **(D)** Visual cluster analysis of cooperation among countries.

From 1985 to 2022, a total of 32 countries performed studies on lipid metabolism in T cells, and the global article productivity is shown in **Figure 3A**. The top 11 with the highest number of outputs were displayed in **Table 1**. Especially, the USA exhibited the highest publications with a total of 123 papers associated with lipid metabolism in T cells over past years. Then, China (n=42) and UK (n=37) took the second and third places, respectively (**Figure 3B**).

**Table 1:** The top 11 productive countries concerning lipid metabolism in T cells.

Rank	Country	Counts	TLCS^1^	TGCS^2^
1	USA	123 (42.4%)	387	12227
2	China	42 (14.5%)	0	512
3	UK	37 (12.8%)	33	2082
4	Germany	28 (9.7%)	50	1415
5	Italy	19 (6.6%)	18	411
6	France	17 (5.9%)	25	826
7	Japan	17 (5.9%)	0	313
8	Switzerland	14 (4.8%)	17	811
9	Canada	11 (3.8%)	59	1646
10	Austria	10 (3.4%)	11	298
11	Sweden	10 (3.4%)	4	158

^1^ TLCS, total local citation score.

^2^ TGCS, total global citation score.

The most cited countries for published researches are the USA (cited 12227 times), followed by UK (cited 2082 times) and Canada (cited 1646 times) (**Figure 3C**). A further co-authorship network of countries/regions with equal to or more than five publications were constructed. The network demonstrated that the cooperation among countries displayed is relatively close. Among them, the USA has been in cooperation with almost all other countries, and Germany possess the most closest cooperation with the USA (**Figure 3D**).

### Analysis of Institutions and Authors

**Figure 4:**
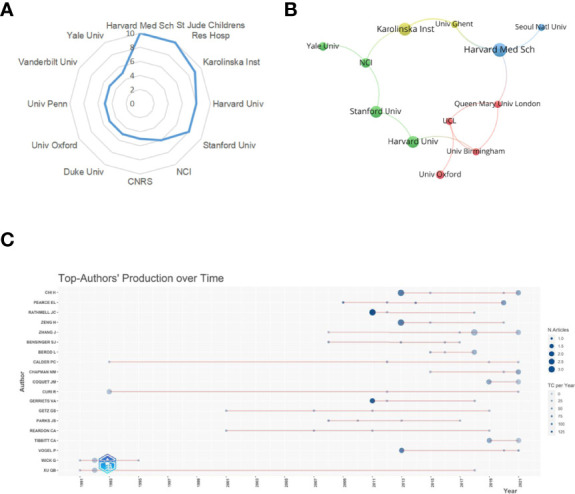
Analysis of institutions and authors. **(A)** Radar map of the top 12 productive institutions; **(B)** Visual cluster analysis of cooperation among institutions. **(C)** The top authors' production over time.

A total of 473 institutions have conducted researches on lipid metabolism in T cells. The top 12 institutions with the most publications are listed in **Table 2**. Among them, Harvard Medical School (n=10) and St Jude Children’s Research Hospital in the USA (n=10) were the leading institutions in terms of outputs, followed by Karolinska Institute in Sweden (n=9) (**Figure 4A**). The institutions with the publication number greater than or equal to four were used to construct co-authorship network. The network demonstrated that the cooperation among institutions presented is relatively not strong, suggesting enhanced cooperation among institutions (**Figure 4B**).

**Table 2:** The top 12 productive institutions concerning lipid metabolism in T cells.

Rank	Institution	Country	Counts	TLCS^1^	TGCS^2^
1	Harvard Medical School	USA	10 (3.4%)	13	670
2	St Jude Children’s Research Hospital	USA	10 (3.4%)	44	826
3	Karolinska Inst	Sweden	9 (3.1%)	4	148
4	Harvard University	USA	8 (2.8%)	5	1473
5	Stanford University	USA	8 (2.8%)	22	1308
6	National Cancer Institute	USA	6 (2.1%)	11	494
7	French National Centre for Scientific Research	French	6 (2.1%)	8	558
8	Duke University	USA	5 (1.7%)	68	1590
9	University Oxford	UK	5 (1.7%)	16	278
10	University Penn	USA	5 (1.7%)	41	1440
11	Vanderbilt University	USA	5 (1.7%)	4	162
12	Yale University	USA	5 (1.7%)	17	492

^1^ TLCS, total local citation score.

^2^ TGCS, total global citation score.

A total of 1896 researchers have published articles on lipid metabolism in T cells till now, and the top 19 productive authors are listed in **Table 3**. Among them, Chi H was the most productive author with 10 publications, followed by Chapman NM (published 6 papers) and Zhang J (published 6 papers). Pearce EL is the most cited author (cited 1751 times), followed by Rathmell JC (cited 1471 times) and Chi H (cited 826 times). Further, a timeline of authors who has had published papers on lipid metabolism in T cells were drawn (**Figure 4C**). Among the top 19 productive authors, Xu QB has keened on this field for more than 30 years since publishing his first paper in 1991; Calder PC and Curi R have keened on this field for about 30 years since publishing their first paper in 1993; Wick G has only published papers between 1991 and 1995; and the other 15 authors engaged in lipid metabolism in T cells after 2001.

**Table 3:** The top 19 productive authors concerning lipid metabolism in T cells.

Rank	Author	Counts	TLCS^1^	TGCS^2^
1	Chi H	10	44	826
2	Chapman NM	6	5	81
3	Zhang J	6	3	52
4	Calder PC	5	0	279
5	Curi R	5	4	94
6	Pearce EL	5	44	1751
7	Zeng H	5	39	736
8	Bensinger SJ	4	76	765
9	Berod L	4	19	303
10	Coquet JM	4	4	95
11	Getz GS	4	4	292
12	Parks JS	4	38	553
13	Rathmell JC	4	67	1471
14	Reardon CA	4	4	292
15	Tibbitt CA	4	4	95
16	Vogel P	4	42	705
17	Wei J	4	5	96
18	WICK G	4	5	45
19	Xu QB	4	5	59

^1^ TLCS, total local citation score.

^2^ TGCS, total global citation score.

### Analysis of Journals

A total of 153 journals accepted studies on lipid metabolism in T cells, and the top 10 core journals are shown in **Table 4**. A total of 83 papers were published in these 10 journals. Frontiers in Immunology (published 19 articles) was the most prolific journal, followed by Journal of Immunology (published 10 articles) and Nature (published 10 articles). Nature was the most cited journal (3137 times), followed by Journal of Immunology (2197 times) and Immunity (1031 times).

**Table 4:** The top 10 core journals publishing lipid metabolism in T cells.

Rank	Journal	Counts	TLCS^1^	TGCS^2^
1	Frontiers in Immunology	19 (6.6%)	0	428
2	Journal of Immunology	10 (3.4%)	88	2197
3	Nature	10 (3.4%)	93	3137
4	International Journal of Molecular Sciences	9 (3.1%)	0	34
5	Immunity	7 (2.4%)	24	1031
6	Cell Reports	6 (2.1%)	9	160
7	Nature Immunology	6 (2.1%)	41	609
8	Proc Natl Acad Sci U S A	6 (2.1%)	35	564
9	European Journal of Immunology	5 (1.7%)	19	269
10	Immunology	5(1.7%)	5	147

^1^ TLCS, total local citation score.

^2^ TGCS, total global citation score.

### Analysis of Cited and Co-Cited References

**Figure 5:**
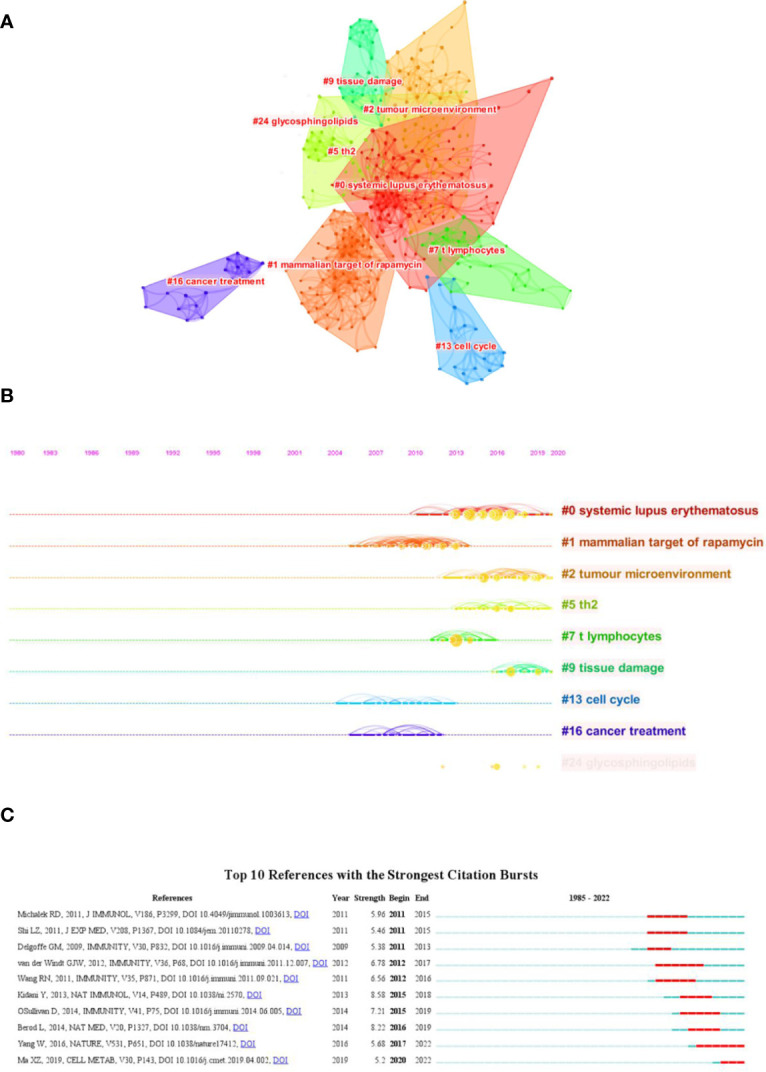
Analysis of cited and co-cited references. **(A)** Cluster analysis of co-cited references; **(B)** Timeline distribution of the top seven clusters; **(C)** Top 10 references with the most strongest citation bursts.

Both cited and co-cited references are foundation for studying the filed researchers interested in, which can provide basic background information. Therefore, we performed an analysis on cited and co-cited references. The top 10 cited references among the 290 publications were presented in **Table 5**, and the top 10 co-cited references was listed in **Table 6**. The article with the most citation and co-citation was written by Michalek RD et al. (77) in 2011 who revealed that lipid oxidation based activation of AMP-activated protein kinase is required for the generation of regulatory T cells (Tregs). Further, we build a co-cited reference network cluster analysis. The modularity Q was 0.8873, and the mean silhouette value was 0.9126, suggesting the excellent quality of the cluster analysis. Nine clusters with the highest K values were plotted (**Figure 5A**), which contain “tumor microenvironment”, “glycosphingolipids”, “th2”, etc. Additionally, we conducted a timeline for these clusters (**Figure 5B**). Relatively, the correlation between lipid metabolism and Th2 cells, and the application of chemicals targeting lipid metabolism in cancer treatment are newly concerned by researchers. Finally, a references burst was performed based on the top 10 co-cited references with the strongest citation (**Figure 5C**). We found that the research published by Kidani et al. (2013) (76) possess the highest bursts strength (8.58), in which they pointed out the necessity of sterol regulatory element-binding proteins (SREBP) in affecting lipid metabolism in activated CD8^+^ T cells. Besides, Yang W had a relatively high citation bursts in recent years.

**Table 5:** The top 10 cited references among the 290 publications.

Rank	First Author	Year	Journal	DOI
1	Michalek RD (77)	2011	Journal of Immunology	10.4049/jimmunol.1003613
2	Pearce EL (10)	2009	Nature	10.1038/nature08097
3	Daynes RA (69)	2002	Nature Review Immunology	10.1038/nri912
4	Patsoukis N (67)	2015	Nature Communications	10.1038/ncomms7692
5	Zeng H (75)	2013	Nature	10.1038/nature12297
6	Daynes RA (69)	2002	Cell	10.1016/j.cell.2008.04.052
7	Patsoukis N (67)	2015	Immunity	10.1016/j.immuni.2014.06.005
8	Daynes RA (69)	2002	Journal of Immunology	10.4049/jimmunol.164.3.1364
9	Patsoukis N (67)	2015	Nature	10.1038/35009119
10	Ma C (71)	2016	Nature	10.1038/nature16969

**Table 6:** The top 10 co-cited publications in lipid metabolism in T cells.

Rank	First Author	Year	Journal	DOI
1	Michalek RD (77)	2011	Journal of Immunology	10.4049/jimmunol.1003613
2	Berod L (79)	2014	Nature Medicine	10.1038/nm.3704
3	Pearce EL (10)	2009	Nature	10.1038/nature08097
4	Kidani Y (76)	2013	Nature Immunology	10.1038/ni.2570
5	O’Sullivan D (68)	2014	Immunity	10.1016/j.immuni.2014.06.005
6	Shi LZ (66)	2011	Journal of experimental medicine	10.1084/jem.20110278
7	Wang RN (64)	2011	Immunity	10.1016/j.immuni.2011.09.021
8	Bensinger SJ (70)	2008	Cell	10.1016/j.cell.2008.04.052
9	van der Windt GJW (65)	2012	Immunity	10.1016/j.immuni.2011.12.007
10	Zeng H (75)	2013	Nature	10.1038/nature12297

### Analysis of Keywords

**Figure 6:**
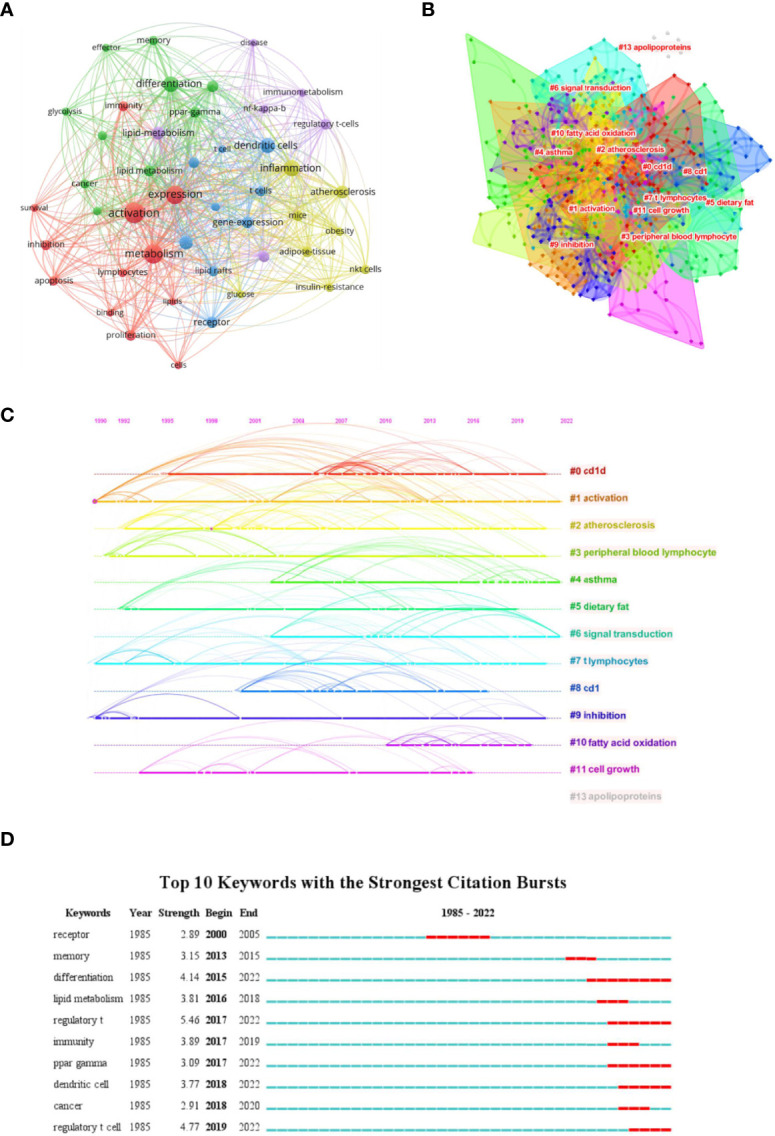
Analysis of Keywords. **(A)** Co-network of keywords; **(B)** Cluster analysis of keywords; **(C)** Timeline distribution of the top 15 clusters; **(D)** Top 10 keywords with the most strongest bursts.

We firstly constructed a network based on extracted keywords (**Figure 6A**). Interestingly, we found the existence of dendritic cells, suggesting the crosstalk between immune cells mediated by lipid. Liu et al. (2019) (74) demonstrated that tregs can enhance the SREBP1-dependent metabolic fitness of macrophages through inhibition of CD8^+^ T cell-released interferon gamma (IFN-γ). However, the crosstalk between T cells and other immune cells is nearly void when discussing lipid metabolism. Thus, studies on exploring this field should be enhanced to fulfill the gap. Subsequently, we built a keywords cluster analysis, and 13 clusters were obtained (**Figure 6B**). The modularity Q and the mean silhouette value of the cluster were 0.624 and 0.8485, respectively, indicating the outstanding quality of this analysis. Based on the clusters, we further plotted a timeline (**Figure 6C**). The timeline of clusters showed that fatty acid oxidation is a relatively new selection for lipids type studied. Finally, the top 10 keywords with the strongest bursts indicated that regulatory T cell (Treg) takes the first place, especially in recent years, suggesting studies on lipid metabolism in regulating tregs attracted the attention of researchers (**Figure 6D**).

## Discussion

In this study, we analyzed the main knowledge domain and emerging trends of lipid metabolism in T cells. Increasing annual outputs and citations of publication were found with time. The USA was the most productive country among the 32 countries, and nine of the top 12 productivity institutions are located in the USA. Among the authors work in the institutions in the USA, Chi H was the most representative. His team mainly concentrated on the effect of lipid metabolism on the differentiation and maintenance of T follicular helper (T-FH) and tregs (72, 73, 75, 78). Their latest study on T-FH cells revealed cytidine diphosphate (CDP)-ethanolamine pathway, along with C-X-C motif chemokine receptor 5 (CXCR5) as essential factors for T-FH cell differentiation (72). Besides, they found that inhibiting SREBPs-depended lipid synthesis in tregs can unleash effective and safe antitumour immune responses, highlighting the potential of SREBPs as checkpoints for tregs (78). Among the top 12 productive institutions, the most cited institution is the Duke University in the USA (published 5 articles, cited 1574 times). The most cited publication in the Duke University is written in 2011 by Rathmell JC who found that CD4^+^ T cell subsets require distinct metabolic programs (77). In detail, they reported that Th1, Th2, and Th17 cells are highly glycolytic due to the highly expressed glucose transporter glucose transporter 1 (Glut1) on the membrane, while low expressive level of Glut1 but high lipid oxidation rates are detected in tregs.

**Figure 7:**
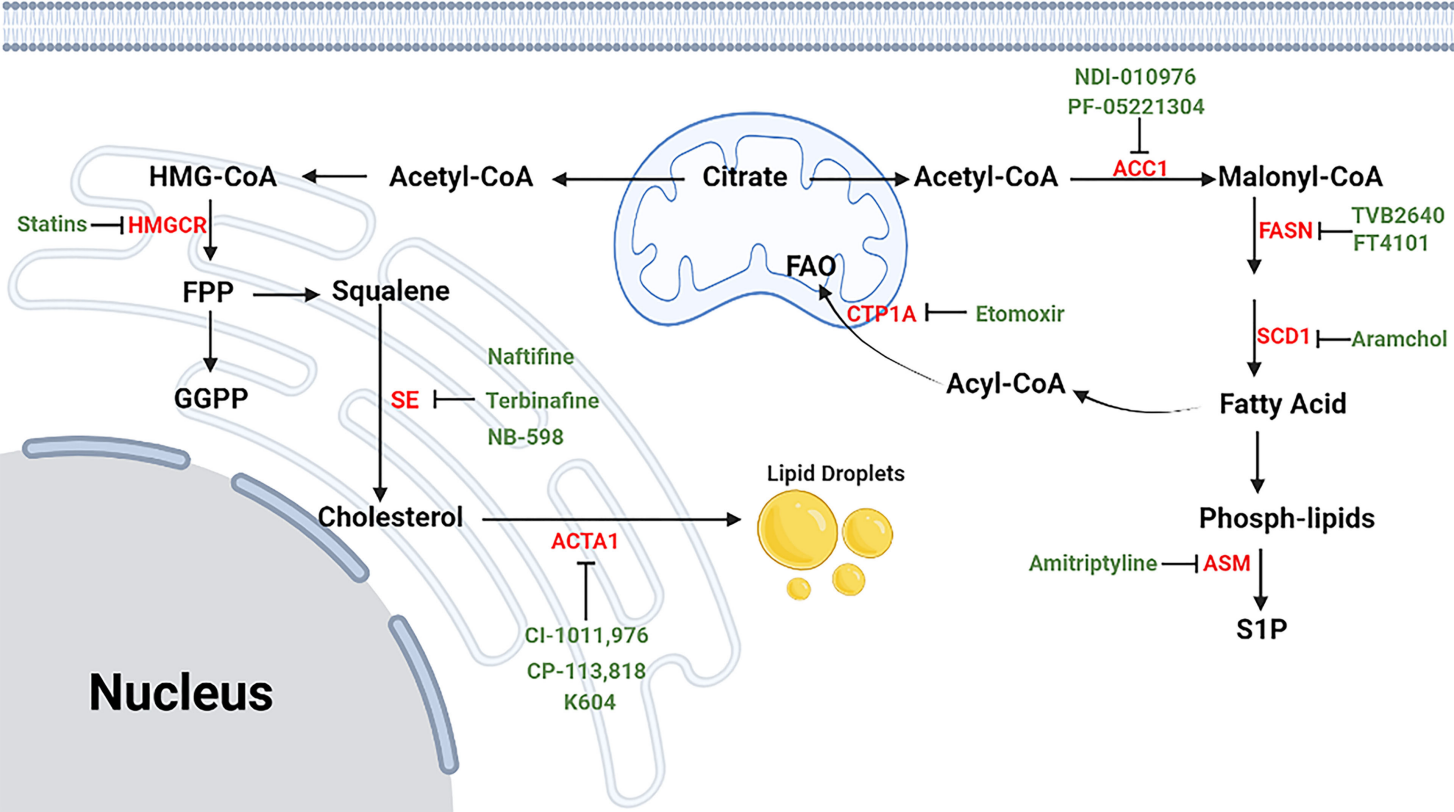
The process of lipid metabolism in T cells. The citrate is exported from mitochondria to cytosol by mitochondrial citrate carriers to generate acetyl-CoA and. Acetyl-CoA is the substrate for the biosynthesis of fatty acid synthesis (FAS), cholesterol (CBP), phospholipids and other active lipids. For CBP, HMG-CoA reductase (HMGCR) is the rate-limiting enzyme mediating the synthesis of acetyl-CoA to farnesyl pyrophosphate (FPP), and statins are specific chemicals inhibiting HMGCR. Squalene monooxygenase (SE) is the rate-limiting enzyme mediating the synthesis from squalene to cholesterol, and naftifine, terbinafine, NB-598 are specific chemicals targeting SE. After cellular distribution, excessive cholesterol is disposed of either through exporting outsides the cells or storing as cholesterol esters *via* cholesterol acyltransferase (ACAT) enzymes, and CI-1011, CI-976, CP-113, CP-818 and K604 are inhibitors of ACAT1. For FAS, Acetyl-CoA is firstly converted into malonyl-COA by acetyl-CoA carboxylase 1 (ACC1), and NDI-010976 and PF-05221304 repress ACC1. Malonyl-COA is further used to synthesize fatty acid through the regulation of FASN ans SCD1. TVB2640 and FT4101 specific target FASN, and specific Aramchol targets stearoyl-Coenzyme A desaturase 1 (SCD1). Fatty acid is synthesized to phospholipids which can hydrolyze to sphingosine-1-phosphate (S1P) through acid sphingomyelinase (ASM), and Amitriptyline suppresses ASM. and it can participate fatty acid oxidation (FAO) after turning into Acyl-COA. Besides, fatty acid can also participate in fatty acid oxidation after entering into mitochondria, which is mediated by CTP1A, and etomoxir is a chemical targeting carnitine palmitoyltransferase 1A (CPT1A).⊣: inhibition.

According to the published documents, lipid metabolism regulates the fate of both CD4^+^ and CD8^+^ T cells (**Table 7**, **Figure 7**). Based on the timelines of references and keywords, and burst of keywords, the differentiation of CD4^+^ T cells, especially tregs, draw large attention recently. Th17 cells are characterized by the secretion of pro-inflammatory cytokine IL-17 to induce inflammatory diseases and tumors, and its differentiation is positively controlled by the nuclear receptor retinoic acid receptor-related orphan receptor γ (RORγt) (41, 42). Accumulation of the cholesterol precursor desmosterol can specifically activate RORγt and subsequently trigger Th17 differentiation (27). Similarly, 7β, 27-dihydroxycholesterol, a derivative oxysterol of cholesterol, was identified as the most selective and potent agonist for RORγt, which can restore the inhibitory effect of ursolic acid (an RORγt inhibitor) on Th17 differentiation (29). In summary, cholesterol uptake and biosynthesis programs are enhanced, whereas cholesterol efflux and metabolism programs are inhibited during Th17 differentiation. Similar effect of cholesterol on tregs differentiation has also been confirmed, and liver kinase B1 (LKB1) was identified as a key regulator. Researchers have proved the mevalonate pathway (the first step of cholesterol synthesis) as the most affected pathway in tregs with specific mTOR deletion (75). Also, tregs with LKB1-deficient showed impaired suppressive activity, which can be restored by the enhancement of the mevalonate pathway (43). Besides, it has been shown that geranylgeranyl pyrophosphate (GGPP), an intermediate metabolites of cholesterol synthesis, amplifies Tregs differentiation *via* enhancing signal transducer and activator of transcription 5 (STAT5) phosphorylation in colitis (24). However, there exists opposite evidence demonstrating a conversed effect of the cholesterol biosynthesis pathway (CBP) on tregs. Researchers showed that targeting SREBPs, a key regulator of cholesterol synthesis, in tregs can unleash its effective anti-tumour responses without autoimmune toxicity (78). The function of other lipids on CD4^+^ T cells has also been studied previously. For instance, naive CD4^+^ T cells with depletion of acid sphingomyelinase (ASM), which is a phospholipids hydrolase enzyme, possess a greater probability to differentiate into Th1 and Th17 cells which can facilitate anti-tumor immunity in non-small-cell lung cancer (31). Besides, Michalek RD et al. (77) revealed that lipid oxidation based activation of AMP-activated protein kinase is required for the generation of Tregs. However, Raud et al. (11) and Saravia et al. (44) argued that fatty acid oxidation medicated by carnitine palmitoyltransferase 1A (CPT1A) is not indispensable to tregs generation or function *in vivo*. Although dispute exists in the effect of lipids on tregs, the importance of them in CD4^+^ T cells differentiation should be attached.

**Table 7:** The effect of part metabolites generated in lipid metabolism on T cells.

Metabolites	Generation Pathway	Key Enzymes	Effect on T cells
FPP^1^/GGPP^2^	Metabolites in CBP	HMGCR^3^	Treg ↑ (24, 25)^13^
			Th1 ↑ (26)
Desmosterol	Precursor of cholesterol	HMGCR^3^	Th17 ↑ (27)
25-hydroxylase	Derivative of cholesterol	CYP25A1^4^	Th1 ↓ (28)^14^
27-hydroxylase	Derivative of cholesterol	CYP27A1^5^	Th17 ↑ (29)
			CD8+ T ↓ (30)
sphingosine-1-phosphate	Hydrolyzate of phospholipids	ASM^6^	Th1 ↓, Th17 ↓ (31)
			Th2 number ↑, activity ↓ (32)
			Treg ↑ (33)
			CD8+ T ↓ (34)
Prostaglandin E2	Prostaglandin	COX^7^, mPGES1^8^	Th1 ↑, Th17 ↑ (35, 36)
			Treg ↑ (37)
			CD8+ T ↑ (38)
Cardiolipin	Glycerophospholipids	GPAM^9^, PTPMT1^10^	CD8+ T ↑ (39)
Platelet activating factor	Glycerophospholipids	GPAM^9^	Th17 ↑ (39)
Long-chain fatty acids	Fatty acid	ACC1^11^, SCD1^12^	Th1 ↑, Th17 ↑ (40)
Short-chain fatty acids	Fatty acid	ACC1^11^, SCD1^12^	Treg ↑ (40)

^1^FPP, farnesyl pyrophosphate; ^2^GGPP, geranylgeranyl pyrophosphate; ^3^HMGCR, HMG-CoA reductase; ^4^CYP25A1, cytochrome P450 family 25 subfamily A member 1; ^5^CYP27A1, cytochrome P450 family 27 subfamily A member 1; ^6^ASM, acid sphingomyelinase; ^7^COX, cytochrome c oxidase; ^8^mPGES1, prostaglandin E synthase; ^9^GPAM, glycerol-3-phosphate acyltransferase, mitochondrial; ^10^PTPMT1, protein tyrosine phosphatase mitochondrial 1; ^11^ACC1, acetyl-CoA carboxylase 1; ^12^SCD1, stearoyl-Coenzyme A desaturase 1. ^13^↑, increased; ^14^↓, decreased.

In addition to CD4^+^ T cells, lipids are also indispensable to CD8^+^ T cells. Cai et al. (45) indicated that activation of RAR related orphan receptor A (RORɑ) with SR1078 (a cholesterol synthetic agonist) can impair the survival and proliferation of activated CD8^+^ T cells. Besides, ablation of cytochrome P450 family 27 subfamily A member 1 (CYP27A1), a rate-limiting enzyme responsible for 27-hydroxycholesterol biosynthesis, could dramatically inhibit metastasis of cancers by decreasing the amount of CD8^+^ T cells (30). These results indicated a positive cholesterol content in CD8^+^ T cells enhances their effect. However, excessive cholesterol in tumor microenvironment was proved to be an inhibitor regulating the anti-tumor activity of CD8^+^ T cells (46). The role of other lipids on CD8^+^ T cells has also been explored previously. For instance, Corrado et al. (39) found that deficiency of cardiolipin-synthesizing enzyme PTPMT1 results in poor response of CD8^+^ T cells to antigens due to the lack of essential cardiolipin. Additionally, Pearce et al. (10) has indicated that the deficiency of long chain fatty acid oxidation (LC-FAO) in T cells leads to defect in their ability to generate long lived memory T (TM) cells, However, Raud et al. (11) demonstrated that LC-FAO is not required for TM cells formation *in vivo*. Besides, Pan et al. (47) reported that specific deficiency of fatty-acid-binding proteins 4 and 5 (FABP4 and FABP5) hampers exogenous free fatty acid uptake by mouse CD8^+^ tissue-resident Tm cells and reduces their survival, while having no effect on the survival of central Tm cells in lymph nodes.

In light of the effect of lipid metabolism on T cells, clearing drugs or inhibitors targeting key enzymes regulating lipid metabolism seems to be helpful (**Table 8**, **Figure 7**). Among these drugs, the cholesterol-lowering chemicals Statin, an inhibitor of HMGCR, is the most widely used. The most recognized role of statin is to promote the differentiation of CD4^+^ T cells into Th2, whereas to inhibit that into Th1 and Th17 in inflammation or autoimmune diseases (49–54). However, Shimada et al. (48) identified statin as a drug inducing Th1 in patients with acute coronary syndrome. As for tregs, the effect of statins is also in dispute. Studies have reported that tregs proliferation, activation and immunological suppressive ability are largely restricted in the presence of simvastatin (75). Opposing data were available regarding the role of statins in patients with rheumatoid arthritis or acute coronary syndrome in which increased number of tregs were detected after treatment with statins (55, 56). For CD8^+^ T cells, statins have been clarified to attenuate T cells exhaustion in subjects with HIV-1 infection (57) and minimize the incidence of tumor recurrence of breast cancer (58). Above evidence suggest the potential value of statins in inflammation, autoimmune diseases and tumors. However, most studies published now are mainly on statins, and statins may elevate the amount and activity of tregs in some patients, which may lead to a terrible outcome for some patients with cancers. Understanding the reason why some effective therapies targeting lipid metabolism function in some patients but not in others, mining new drugs targeting other key enzymes, and exploiting the combined use of drugs targeting lipid metabolism with immunotherapy, radiotherapy or chemotherapy are our future research direction.

**Table 8:** The effect of part drugs targeting key enzymes in lipid metabolism on T cells.

Chemicals	Targeted enzymes	Effect on T cells
Statin	HMGCR^1^	Th1 ↑ (48)^7^
		Th1 ↓ (49–51)^8^
		Th17 ↓ (51, 52)
		Th2 ↑ (53, 54)
		Treg ↑ (55, 56)
		Treg ↓ (75)
		CD8+ T ↑ (57, 58)
Avasimibe	ACAT1^2^	CD8+ T ↑ (59, 60)
PF-05221304	ACC1^3^	Th1 ↓, Th17 ↓ (61)
Soraphen A	ACC1^3^	Th17 ↓ (79)
		Treg ↑ (79)
Amitriptyline	ASM^4^	Th2 number ↑, activity ↓ (32)
		Treg ↑ (33)
		CD8+ T ↓ (34)
Etomoxir	CPT1A^5^	Th17 ↓ (62)
		Treg ↑ (11)
		${\text{T}}_{M}^{6}$ formation ↑ (11)
		CD8+ T ↓ (63)

^1^HMGCR, HMG-CoA reductase; ^4^ACAT1, acetyl-CoA acetyltransferase 1; ^3^ACC1, acetyl-CoA carboxylase 1; ^4^ASM, acid sphingomyelinase; ^5^CPT1A, carnitine palmitoyltransferase 1A; ^6^T_M_, memory T cells; ^7^↑, increased; ^8^↓, decreased.

Although we summarized the past and looked to the trends in further in the field of lipid metabolism in T cells through a relatively comprehensive bibliometrics analysis, there still exist several limitations. First, despite our efforts to obtain the most comprehensive literature, some articles were still not included in the analysis, which may cause bias in the analysis. Second, some of the studies with excellent quality published in recent years may failed to obtain excessive citations due to the limited time span, they had not been highlighted in this analysis. Thirdly, this project is performed based on machine algorithm, which may lead to a slightly insufficient evidence.

Our results indicated that researches on lipid metabolism in T cells are developing rapidly at present. The USA is a major producing country, which generates many breakthroughs in this field. Frontiers in Immunology and Nature were the most prolific and cited journals, respectively. Finally, the correlation between lipid metabolism and Th17 and Tregs cells attracted increasing attention recently.

## Data Availability Statement

The original contributions presented in the study are included in the article/**Supplementary Material**. Further inquiries can be directed to the corresponding authors.

## Author Contributions

PC, CZ, and SJ performed this bibliomrtrics analysis and wrote manuscript. YZ, YL, QX, and JC participated in experimental design and manuscript writing. HL and XF designed this study and organized the manuscript writing. All authors contributed to the article and approved the submitted version.

## Funding

This work was supported by the National Key Research and Development Program (grant number 2016YFC0906400) and China’s National Natural Science Foundation (grant number 81874059).

## Conflict of Interest

The authors declare that the research was conducted in the absence of any commercial or financial relationships that could be construed as a potential conflict of interest.

## Publisher’s Note

All claims expressed in this article are solely those of the authors and do not necessarily represent those of their affiliated organizations, or those of the publisher, the editors and the reviewers. Any product that may be evaluated in this article, or claim that may be made by its manufacturer, is not guaranteed or endorsed by the publisher.
